# Vegetable Oils Rich in Polyunsaturated Fatty Acids Supplementation of Dairy Cows’ Diets: Effects on Productive and Reproductive Performance

**DOI:** 10.3390/ani9050205

**Published:** 2019-04-30

**Authors:** Teresa Castro, Diego Martinez, Beatriz Isabel, Almudena Cabezas, Vicente Jimeno

**Affiliations:** 1 Faculty of Veterinary, Universidad Complutense de Madrid, 20840 Madrid, Spain; bisabelr@pdi.ucm.es (B.I.); almucabe@ucm.es (A.C.); 2 Kemin Animal Nutrition and Health-EMENA, 2200 Herentals, Belgium; diego.martinez@kemin.com; 3 School of Agricultural, Food and Biosystems Engineering, Polytechnic. University of Madrid, 28040 Madrid, Spain; vicente.jimeno@upm.es

**Keywords:** dairy cows, polyunsaturated fatty acid, soybean oil, linseed oil, reproduction

## Abstract

**Simple Summary:**

Ruminants milk contains some bioactive lipids that have a beneficial effect on human health. The present study aimed to evaluate the benefit of incorporating polyunsaturated fatty acids rich vegetable oils on productive and reproductive performance of dairy cows. The results show that including polyunsaturated fatty acids and rich vegetable oils in rations of dairy cows improve the nutritional profile of milk and some reproductive parameters. Ruminant milk often has a negative image for health because of its fat content and its composition. A way to improve the nutritional profile of the milk is to supplement dairy cows’ diets with polyunsaturated vegetable oils, which makes it healthier for the consumer and improves the commercial value of the milk in view of the continued decline in fertility among dairy cows. The possibility of supplementing the diet with vegetable oils rich in polyunsaturated fatty acids as a means of improving reproductive performance has considerable interest for dairy producers.

**Abstract:**

The aim of this study was to determine how polyunsaturated fatty acids (PUFA) supplementation can affect the productive and reproductive performance in dairy cows subjected to a fixed-time artificial insemination (TAI) protocol under farm conditions. One hundred and ninety-eight Holstein non-pregnant cows were used. Treatments consisted of a control diet (CON), without added oil, and two diets supplemented with either 2.3% soybean oil (SOY) or 2.3% linseed oil (LIN) as dry matter. The diets were formulated to be isoenergetic and isoproteic. Dry matter intake and milk yield were similar among treatments (*p* > 0.05). Both the percentage of fat (*p* = 0.011) and protein (*p* = 0.022) were higher in milk from animals not fed with oil (CON). The greatest saturated fatty acid (SFA) concentration (*p* < 0.0001) was observed in milk from cows fed the control diet, without added oil. The monounsaturated fatty acids (MUFA), PUFA, and the n-3 PUFA content was higher (*p* < 0.0001) in the milk from animals fed with oil with respect to the control treatment. The C18:2 cis-9, trans-11 in the milk of animals fed with oil supplements was significantly higher (*p* < 0.0001) than in that of the control group. Animals supplemented with linseed oil tended to show higher plasma progesterone level (*p* = 0.09) and a higher number of pregnant cows on the first artificial insemination (*p* = 0.07). These animals tended to reduce the number of TAI (*p* = 0.08). In brief, results showed that vegetable oils rich in PUFA supplementation considerably improve the nutritional profile of milk. PUFA n-3 supplementation slightly improves some reproductive parameters in dairy cows subjected to the fixed-time artificial insemination (TAI) protocol.

## 1. Introduction

Reproductive success is fundamental for the profitability and sustainability of dairy farms that use confined total mixed ration (TMR)-based systems. One of the main economic drawbacks of these systems is reproductive inefficiency, particularly during the negative energy balance phase that occurs at the beginning of lactation. Supplementing the diet with fats to help improve the energy status, and, therefore, reproductive performance, is common practice. However, fats have been shown to have a positive effect on reproductive performance in many cases, irrespective of the cow’s energy status [1]. Supplementation usually consists of saturated fats in order to avoid possible alterations in the animal’s ruminal metabolism. However, it has been suggested that long-chain fatty acids (LCFA), particularly polyunsaturated fatty acids (PUFA), improve reproductive performance [2]. Therefore, in view of the continued decline in fertility among dairy cows, the possibility of supplementing diet with PUFA as a means for improving reproductive performance has aroused considerable interest. 

The main fatty acids of interest in cattle reproduction are arachidonic acid (C20:4n-6) and eicosapentaenoic acid (C20:5n-3). These LCFA are synthesized from linoleic acid (C18:2n-6) and α-linolenic (C18:3n-3) in a series of stages involving desaturation and elongation [3]. Although the percentage of linoleic (C18:2n-6) and α-linolenic acid (C18:3n-3) converted to long-chain PUFA is quite low, there is evidence that the concentration of n-3 in plasma [4], red blood cells [5], meat [6], milk [7], and reproductive tissue [8] is determined by the concentrations of dietary linoleic (C18:2n-6) and α-linolenic (C18:3n-3). Both arachidonic acid (C20:4n-6) and eicosapentaenoic acid (C20:5n-3) are precursors of prostaglandins, but prostaglandins synthesized from eicosapentaenoic acid (C20:5n-3) do not share the same biological activity as those produced from arachidonic acid (C20:4n-6) [3].

Ruminants milk contains some bioactive lipids, such as C4:0, odd and branched chain fatty acids, C18:1, cis-9, and the conjugated linoleic acid (CLA) C18:2 cis-9, trans-11. All of these entities have a beneficial effect on human health and prevent the onset and development of some chronic diseases [9,10]. The development of feeding systems designed to produce milk that is low in medium chain saturated fatty acids and high in odd and branched chain fatty acids, C18:1, cis-9, and C18:2 cis-9, trans-11 CLA and PUFA could have long-term benefits on human health, without the need for consumers to change their dietary habits. In ruminant diets, the main source of α-linolenic acid (C18:3n-3) is forage and linseed oil, while linoleic acid (C18:2n-6) is found in the fat of most grains and of soybeans, safflower seed, and sunflower seed. Vegetable oils obtained from these oilseeds (soybean oil, safflower oil, sunflower oil, or linseed oil), can be added to rations in the form of the seed itself, or as free oils. The most widely used vegetable oil is soybean oil because of its greater availability in commodity markets, which is followed by sunflower or linseed oil. In recent years, interest has been re-kindled in linseed as a means of modifying the fatty acid content of milk and improving the reproduction performance in cows [11,12]. Most studies in oils rich in PUFA have involved oilseeds and fat levels above those usually found in TMR. However, very few studies have compared diets with no added fat against diets supplemented with free vegetable oils at normal levels of inclusion in TMR. 

Currently, a large majority of farms use TAI synchronization protocols due to the difficulty in detecting estrus. However, conception rates are generally lower than those obtained with heat detection protocols. Therefore, it would be of great interest to develop feeding strategies for TAI protocols that would improve reproduction rates.

The objective of this experiment conducted under farm conditions has been to study the benefit of incorporating PUFA-rich vegetable oils at normal levels in TMR on productive performance, and their effect on the reproductive performance of dairy cows subject to fixed-time artificial insemination protocols.

## 2. Materials and Methods 

### 2.1. Animal and Experimental Diets

The study was carried out on a commercial farm (Calidad Pascual Experimental Farm) located in Fuente Espina (Burgos, Spain). A total of 198 non pregnant Holstein dairy cows (114 multiparous and 84 primiparous) were used. The animals were divided into six batches, according to milk production and parity, and were randomized to the different experimental treatments (two replicas per treatment). All animal handling practices followed the Spanish Policy for Animal Protection RD53/2013, which meets the European Union Directive 2010/63/UE about the protection of animals used for scientific purposes. Treatment consisted of a control diet with no added oil (CON) and two diets supplemented with either 2.3% soybean oil (SOY) or 2.3% linseed oil (LIN) as dry matter. Experimental rations were supplied as TMR with a forage: concentrate ratio of 40:60, and were formulated to be isoenergetic and isoproteic, according to the Institute National de la Recherche Agronomique guidelines [13] (Table 1).

The fatty acid profile of the experimental diets and oil supplements are shown in Table 2.

### 2.2. Reproductive Management

Presynch was started at 32 ± 3 days postpartum (ppd), with the administration of two injections of PGF_2α_ (Dinolytic^®^, 5 mg of dinoprost tromethamine, Pfizer Animal Health, Madrid, Spain) at a 14-day interval (32 ± 3 and 46 ± 3 ppd). Ovsynch was started 11 days after the second injection of PGF_2α_, with administration of an injection of GnRH (57 ± 3 ppd) (Fertagyl^®^, gonadorelin 0.1 mg, Veterinaria Esteve, Barcelona, Spain). Seven days after the injection of GnRH, a third injection of PGF_2α_ (64 ± 3 ppd) was administered, and two days thereafter (66 ± 3 ppd) a second injection of GnRH was given. All cows underwent TAI approximately 18 hours after the second injection of GnRH. Pregnancy was diagnosed at 28 days, and was confirmed with a transrectal ultrasound scan (Ecogra, 7.5 MHz transrectal linear transducer; Aloca Co., Ltd., Tokyo, Japan) 60 days after the first TAI. A new Ovsynch ovulation synchronization protocol was initiated in non-pregnant animals. This started on the day the pregnancy diagnosis was made. The animals were re-inseminated following the same guidelines.

### 2.3. Experimental Procedure

The cows were housed in six free stall barns. Experimental rations were given for at least 32 weeks, starting two days after delivery. The quantity of food offered and the amount of food not consumed were weighed in each batch on a daily basis. The amount of ration offered in each batch was calculated from the previous day’s consumption, in order to maintain a 5% non-consumed food margin. The dry matter intake (DMI) in each batch was divided by the number of cows in the batch to estimate DMI per cow per day. Monthly samples were taken of TMR. These were frozen at −24 °C for laboratory analysis.

**Figure 1:**
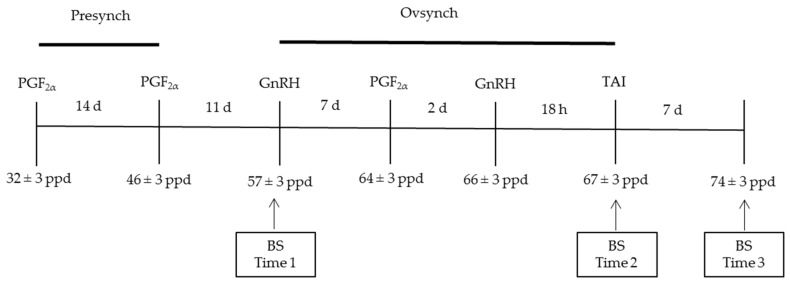
Diagram of blood samples and reproductive management. TAI = fixed-time artificial insemination, ppd = postpartum day, and BS = blood sample collection.

Blood samples were extracted from the coccygeal vein and collected in Vacutainer tubes (BD Vacutainer Systems, Plymouth, United Kingdom) with no anticoagulants. The blood was allowed to clot for approximately 12 h and then centrifuged for 15 min at 3500 rpm. The plasma was transferred to an eppendorf tube and frozen at −20 °C for laboratory determination of progesterone, β-hydroxybutyrate (BHBA), non-esterified fatty acid (NEFA), insulin, and insulin-like growth factor-1 (IGF-I) concentrations. Blood samples were collected at 57 ppd, 67 ppd, and 74 ppd (Figure 1).

The reproductive data were collected over the study period, and the following parameters were calculated: number of pregnant cows to first TAI, number of pregnant cows, number of TAI, and open days (days from calving to confirmed pregnancy).

Daily milk yield from each cow was recorded for 120 days (from 30 DIM to 150 DIM). For the milk analysis, eight animals were selected from each batch (16 animals per treatment) from which a weekly sample was obtained for three weeks (100 DIM, 107 DIM, and 114 DIM). The samples were taken from the morning and evening milking session, and were proportional to the amount of milk produced in each session. Milk samples taken from each milking session were combined and divided into two batches. One was stored at 4 °C until the fat and protein analyses were performed. The other was stored at −24 °C and was used to determine fatty acids.

### 2.4. Analytical Procedure

Samples of TRM were analyzed for dry matter (DM) (AOAC official method 934.01), ash (AOAC official method 942.05), Kjeldahl nitrogen (AOAC official method 941.04), and ether extracts (AOAC official method 920.39). Neutral detergent fiber (NDF) and acid detergent fiber (ADF) analyses were carried out as described by Van Soest et al. [14], using the ANKOM^200/220^ Fiber Analyser (ANKOM Technology Corp., Fairport, NY, USA). NDF analysis was performed with sodium sulphite and alpha amylase. The starch content was determined by the Ewers polarimetric method [15]. Fat and protein content in milk were determined in accordance with the International Dairy Federation [16], using a MilkoScan analyzer (FOSS Electric A/S, Hillerød, Demark).

Determination of fatty acid content in feed was performed, according to the One Step extraction and quantification procedure proposed by Sukhija and Palmquist [17]. Methyl esters of fatty acids were analyzed by gas chromatography using a Hewlett-Packard HP-6890 (Avondale, PA, USA) gas cromatograph equipped with a flame ionization detector and a capillary column HP-Innowax (100 × 0.32 mm × 0.25 film thickness, polyethylene glycol), as described by Kramer et al. [18]. Milk fat from freeze-dried milk was extracted using a mixture of chloroform/methanol (2:1, *v*/*v*) following the procedure described by Folch et al. [19]. Fat extracts were methylated in the presence of sodium methoxide, according to the method proposed by Christie [20] with modifications. Hexane (2 mL) was added to 40 mg of fat extracts followed by 40 μL of methyl acetate. After the mixture was vortexed, 40 μL of methylation reagent (1.75 mL methanol:0.4 mL of 5.4 mol/L sodium methylate) was added. The mixture was vortexed and allowed to react for 10 min. Then 60 μL of termination regent (1 g oxalic acid/30 mL diethyl ether) was added. The sample was then centrifuged for 5 min at 2400× *g* at 5 °C leaving a clear layer of hexane. An aliquot of the hexane was taken and used directly for chromatographic determination described for the fatty acids from the feed.

Plasma concentrations of NEFA were determined by the enzymatic colorimetric method (ACS-ACOD-MEHA, NEFA C, Wako^®^, Neuss, Japan), using an Olympus AU400 analyser (Olympus Diagnostica GmbH, Hamburg, Germany). A competitive enzyme immunoassay (ELISA, EIA-1561, DRG Diagnostics, Marburg, Germany) determined plasma concentrations of progesterone. Plasma concentrations of insulin were determined by sandwich ELISA (Mercodia Bovine Insulin ELISA, Sweden), using the EMS Reader MF V.2.9-0. Similarly, sandwich ELISA (Active Non-Extraction IGF-1 ELISA DSL-10-2800, Diagnostic Systems Laboratories Inc., Webster, TX, USA) determined IGF-1 concentrations. Lastly, D-3-Hydroxybutyrate concentrations were determined by enzyme kinetics, using the RANBUT D-3-Hydroxybutirate (RANDOX^®^) reagent (Crumlin, UK).

### 2.5. Statistical Analysis

Statistical analysis was performed using the Statistical Analysis System (SAS) package V 9.4.

Milk yield, composition, fatty acid content, and blood parameters were analyzed as repeated measures using the PROC MIXED using the following model:

Y_ijkl_ = µ + D_i_ + T_j_ + C_k_ + DT_ij_ + ε_ijkl_ (1)

where Y_ijkl_ = the dependent variable, µ = the overall mean, D_i_ =the effect of diet, T_j_ = the effect of time of sampling, C_k_ = the effect of cow, DT_ij_ = the interactions between diet (D) and time (T), and ε_ijkl_ = the random residual error. In the model, the diet (D) was the main effect, the cow (C) was the random effect, and the time of sampling (T) was the repeated measure. The covariance structure that best fitted the data was chosen based on the Schwartz Bayesian Information Criterion (BIC). The covariance structure with the lowest BIC value from among the following structures: compound symmetry (CS), first-order auto-regressive (AR1), heterogenous first-order autoregressive (ARH1), and unstructured (UN) was taken as the best fit. All four structures showed convergence, and, in all cases, the CS structure was selected since it presented the lowest BIC. The level of significance was established at *p* < 0.05, but *p* < 0.1 was considered a trend.

Number of pregnant cows to first TAI and number of pregnant cows were analyzed using the Chi-square test (χ2) with PROC FREQ. The test was compared the effect of diet, which compared the pregnant cow to the first TAI and the pregnant cows against the non-pregnant cows to the first TAI. This allowed us to compare all possible combinations of dietary treatment pairs. The number of TAI and open days were analyzed using the Kruskal-Walis test with PROC NPAR1WAY. The three groups were compared two by two with the Mann-Whitney W test (Wilcoxon) to compare medians. The Dunn–Sidák multiple comparison method [21] was performed for *p*-values obtained from all the comparisons studied.

## 3. Results

Four cows were diagnosed with mastitis, so the data obtained from those animals were not used in the final analysis.

### 3.1. Effects of PUFA Supplementation on Productive Performance

During the experimental period, the DMI averaged 23.9 kg/d. Milk yield was not significantly affected by dietary treatments (Table 3). However, milk from cows fed with PUFA-rich oils (SOY and LIN diets) had lower concentrations of fat (*p* = 0.011) and protein (*p* = 0.022) than cows fed the CON diet. No differences were observed between the SOY and LIN diets.

The FA content in milk is shown in Table 4. Supplementary soybean oil and linseed oil (SOY and LIN diets) reduced the overall proportion of saturated fatty acids (SFA) (*p* < 0.0001), increased the monounsaturated fatty acids (MUFA) (*p* < 0.001) and PUFA (*p* < 0.0001) content, decreased the proportion of C4:0 (*p* = 0.002), C12:0 (*p* = 0.039), and C16:0 (*p* < 0.0001), and increased that of C18:0 (*p* = 0.02) compared to the diet without added oil (CON diet). 

Different proportions (*p* = 0.004) of oleic acid (C18:1 cis-9) were found in each experimental diet (20.06%, 26.24%, and 23.20% in the CON, SOY, and LIN diets, respectively). Supplementary soybean (SOY) and linseed (LIN) oil increased (*p* < 0.0001) the linoleic acid (C18:2 cis-9, cis-12) and α-linolenic acid (C18:3 cis-9, cis-12, cis-15) content compared to the diet with no supplementary oil (CON). 

The increase in C18:2 cis-9, cis-12 was higher in cows receiving the SOY diet compared with the LIN diet, while those receiving the LIN diet showed a higher proportion of C18:3 cis-9, cis-12, and cis-15 compared to the SOY diet. Despite the significant increase in these fatty acids compared with the CON diet, their content in milk was relatively low due to the intense biohydrogenation of unsaturated fatty acids in the rumen.

Supplementation with oils rich in PUFA, both soybean (SOY) rich in C18:2, and linseed (LIN) rich in C18:3, increased the C18:1 trans-11 and C18:2 cis-9, trans-11 content. C18:1 trans-11 acid increased by 16% and 122%, and C18:2 cis-9, trans-11 increased by 41% and 55% in the SOY and LIN diets, respectively, compared with the CON diet. The desaturation index, calculated according to Kelsey et al. [22], as the relationship between the product and the sum of the product and the substrate, was used as a possible indicator of Δ^9^ desaturase activity. The ruminant milk contains four products, which includes C14:1, C16:1, C18:1 cis-9 and C18:2 cis-9 trans-11. These originate from desaturation of C14:0, C16:0, C18:0, and C18:1 trans-11 by this enzyme, respectively. Of these, the best indicator of Δ9 desaturase activity is the correlation between C14:1 and C14:0, since, in milk fat, all C14:0 is produced by de novo synthesis in the mammary gland, and, therefore, desaturation is the only source of C14:1 [23]. The results of our study show higher proportions in the milk of cows fed with the SOY diet compared to the LIN diet, which suggests a greater activity of this enzyme in the mammary gland of these animals.

The atherogenicity index was calculated according to Ulbricht and Southgate [24] as the relation between the proportion of fatty acids capable of increasing serum cholesterol levels (lauric, C12:0, myristic, C14:0 and palmitic acids, C16:0) and protective fatty acids (MUFA and PUFA) in milk (C12:0 + 4 × C14:0 + C16:0)/(MUFA + PUFA). According to the results obtained, the index of the milk of cows fed with the SOY or LIN diets was lower (*p* < 0.0001) than those without oils (CON).

### 3.2. Effects of PUFA Supplementation on Reproductive Performance

The cows that received the LIN diet tended to show a higher (*p* = 0.07) number of pregnant cows in the first TAI and a reduction in the number of TAI (*p* = 0.08) compared to those fed the CON diet (41.5% vs. 23.1% and 2.3% vs. 2.8% mean number of TAI). Animals that consumed the SOY diet showed intermediate values (35.9% and 2.4%) (Table 5). The number of pregnant cows was also higher in the group receiving the LIN diets compared to the CON group, but the differences were not statistically significant (*p* > 0.05) (Table 5).

Table 6 shows the effects of diet (D), time of sampling (T), and D × T interaction on plasma concentrations of progesterone, BHBA, NEFA, insulin, and IGF-1. No statistically significant interaction was observed in any of the variables studied.

Diet tended to increase plasma concentrations of progesterone (*p* = 0.09), increased plasma concentrations of NEFA (*p* = 0.04), decreased plasma concentrations of IGF-1 (*p* = 0.002), and did not affect plasma levels of BHBA or insulin (*p* > 0.05).

Total plasma progesterone levels were higher in cows fed the LIN diet compared to the CON diet (4.03 vs. 3.10 ng/mL). The plasma concentration of NEFA was higher in diets supplemented with oil (0.36 and 0.35 mmol/L in SOY and LIN diet vs. 0.31 mmol/L in the con diet).

The highest IGF-1 values were observed in the CON diet (161.89 ng/mL), followed by SOY (149.02 ng/mL) and LIN (130.60 ng/mL).

## 4. Discussion

In dairy cows, supplementing TMR with 2.3% of PUFA-rich oils (soybean and linseed) did not affect milk yield when compared to the cows receiving the control diet (no oil). This suggests that the addition of free oils did not negatively affect food intake, and, therefore, had no effect on the milk yield. The consumption of large amounts of vegetable oils is usually associated with a negative effect on ruminal digestion, particularly structural carbohydrates [25]. Our results are consistent with those of many published studies including some that are administered higher proportions of free oils than those used in our study [26,27]. Other studies, in contrast, report a negative effect [28], and still others report a positive effect when the diet is supplemented with such unsaturated oils as fish oil [29] or linseed oil [30]. According to Palmquist and Jenkins [31], the forage:concentrate ratio (F:C) appears to influence the response to oil supplementation. Ueda et al. [30], in a study on the effects of supplementing a forage-rich diet (F:C, 65:35) or a concentrate-rich diet (F:C, 35:65) with linseed oil (0% and 3%) on ruminal digestion, observed a significant interaction between the effect of the oil and the forage:concentrate ratio of the ration. This shows that the effect of linseed oil supplementation on ruminal fiber digestibility is positive in rations with a high forage content, but negative in rations with high concentrate or starch content. In our study, the percentage of added oil (2.3% DM) and the forage:concentrate ratio (40:60) may have minimized the effects of fat on ruminal digestion, and, therefore, had no negative effect on DMI or milk yield. 

Supplementation with PUFA-rich oils reduced (*p* < 0.05) the percentage of fat and protein in the milk. Supplementary fat decreases de novo synthesis in the mammary gland [32]. This could be due to reduced ruminal synthesis of acetate and butyrate, or the inhibition of lipogenic enzymes by certain fatty acids produced during the biohydrogenation of PUFA [32], under certain ruminal fermentation conditions [33]. In our study, the addition of PUFA-rich oils could have reduced lipogenesis in the mammary gland and, thus, reduced the fat content in milk. This coincides with the changes observed in the fatty acid profile of the milk, particularly the increase in C18:2 trans-10 cis-12 CLA content in rations containing soybean and linseed oil (Table 4). Although the main source of de novo synthesis inhibition is ruminal biohydrogenation of C18:2n-6 (mainly the trans-10, cis-12 isomer), other studies have shown that the addition of free oils rich in C18:3n-3 can also produce trans isomers involved in the inhibition of de novo synthesis. This induces a response similar to that of oils rich in C18:2n-6 [34].

The results of studies in supplementary polyunsaturated oils and milk protein vary greatly, with some authors observing an increase [23,27], others observing no change [35], and still others observing a reduction [36,37] in protein percentages. Coinciding with our results, they generally show that reducing the percentage of protein does not generally affect [38], or can even increase [37] the amount of protein. According to some authors, the reduction in the protein content of milk is due to changes in ruminal fermentation, which reduce the synthesis of microbial protein [38] or to a dilution effect [39]. In our study, protein content decreased, even though milk yield did not increase. De Peters and Cant [40] indicate that the dilution effect only partially explains the reduction in protein content, and studies such as ours, in which supplementary fat does not affect milk yield but reduces the percentage of protein, would support this affirmation.

In line with our results, most studies in dairy cattle show that giving supplementary PUFA-rich oils reduces short and medium chain fatty acids (SMCFA) content and increases C18s [26,32,41]. As a result, supplementary oils increase the transport of long-chain fatty acids absorbed in the gut to the mammary glands, and reduce de novo synthesis. Even though unsaturated fatty acids undergo ruminal hydrogenation, adding them in free form to the diet increases their levels in milk and inhibits the synthesis of SMCFA in the mammary gland [42]. Our results corroborate these findings, since, in our study, higher percentages of stearic acid (C18:0) were found in the milk fat of animals supplemented with oil, irrespective of the type (soy or linseed). However, higher percentages of oleic acid (C18:1 cis-9) were found in the milk of cows that consumed soybean oil, which could be associated with higher Δ^9^ desaturase index observed in the soybean oil compared to the linseed oil diet. Bu et al. [41], in a study very similar to ours, observed no differences in this index when comparing three rations: control (no added fat), soybean oil, and linseed oil. 

Many studies corroborate our findings that supplementing rations with oils rich in linoleic (C18:2n-6) or α-linolenic acid (C18:3n-3) increases C18:1 trans-11 and C18:2 cis-9, trans-11 content [11,27,41,43]. The amount of C18:2 cis-9, trans-11 CLA in milk depends on the amounts of C18:2 cis -9, trans-11 CLA and C18:1 trans-11 produced in the rumen, together with the activity of Δ^9^ desaturase in mammary tissue. In our study, the increase observed in C18:1 trans-11 and C18: 2 cis-9, trans-11 content in the milk of the animals supplemented with linseed oil compared to those consuming soybean oil does not coincide with the levels reported by Bu et al. [41]. These authors, when supplementing with 4% soy or linseed oil, obtained a greater increase in C18:1 trans-11 and C18:2 cis-9, trans-11 content with soybean than with linseed (an increase of 318% and 105% in C18:1 trans-11 content, and of 273% and 150% in C18:2 cis-9, trans-11 content in rations with soy or linseed, respectively) compared to controls (no fat). In their discussion, the authors attribute this to the more thorough ruminal biohydrogenation of unsaturated fatty acids in cows fed with linseed. Doreau and Ferlay [44], however, found no correlation between linoleic acid (C18:2n-6) levels and the degree of ruminal biohydrogenation. Our study differs from Bu et al. [41] in the amount of oil added (4% of the total MS in Bu et al. [41] compared to 2.3% in ours) and the forage: concentrate ratio (50:50 versus 40:60). Moreover, changes in rations, particularly those associated with milk fat depression, are known cause changes in the microbial population, and these could alter ruminal biohydrogenation of MUFA and PUFA as well as promote the formation of other isomers [45]. This would occur in rations with high levels of concentrate or fat. Capoprese et al. [43], meanwhile, show that endogenous synthesis of C18:2 cis-9, trans-11 CLA by Δ^9^ desaturase activity in the mammary gland correlates closely with C18:1 trans-11 content, and Fievez et al. [46] observed that changes in the concentration of C18:2 cis-9, trans-11 CLA depend fundamentally on the content of C18:1 trans-11 and, to a lesser extent, on Δ^9^ desaturase activity. Our results, like those of numerous other studies [41,43], show that high levels of C18:1 trans-11 acid are associated with high levels of C18:2 cis-9, trans-11 CLA in milk.

One of the main objectives of this study has been to evaluate the effects of different types of vegetable oils on the composition of fat, in order to obtain milk that is more beneficial to human health. In this regard, the milk of the animals fed with soybean or linseed oil has a better fatty acid profile than what was obtained from the control group, due to a higher C18:2 cis-9, trans-11 CLA, and C18:1 trans-11 content, reduced levels of SMCFA and saturated fatty acids, increased MUFA and PUFA levels, and higher n-3 series fatty acids content. Due to the capacity of Δ^9^ desaturase in human tissue to transform C18:1 trans-11 into C18:2 cis-9, trans-11 CLA [47], the increased C18:1 trans-11 content in milk improves its lipid profile. According to the atherogenicity index obtained, milk from cows that consumed soybean oil or linseed was better than milk from those that did not consume oil.

The greater total plasma concentration of progesterone observed in cows supplemented with linseed oil compared to the control diet (no oil) is consistent with the positive influence of n-3 PUFA on circulating levels of progesterone [48,49]. In addition, cows supplemented with linseed, which have the highest plasma progesterone concentration, showed improvement in some reproductive performance indices (number of pregnant cows to first TAI and number of TAI). Several studies have reported improvements in the conception rate of milk cows with high levels of circulating progesterone in TAI protocols [50,51]. Ambrose et al. [52] observed a higher concentration of progesterone at the time of insemination, which is a trend toward a higher conception rate in the first TAI, and lower gestation losses in cows fed with linseed compared to those given sunflower seed, which resulted in a higher proportion of pregnant cows. They conclude that secretion of PG2α could have been attenuated at the time of pregnancy recognition in cows given linseed. Stronge et al. [53] showed that low levels of progesterone between days five and seven after insemination was associated with low fertility in dairy cows. Man et al. [54] observed that intra-vaginal supplementation with progesterone five days after insemination resulted in better embryo development. Petit and Twagiramungu [55] observed lower gestational losses with supplementary linseed compared to a diet containing calcium soap of palm oil (Megalac) and another with micronized soybeans, as a result of their modulatory effect on progesterone levels and the size of the corpus luteum. All these studies show the importance of progesterone levels in the first days after insemination, which can be one of the factors that determine the success or failure of pregnancy in dairy cows.

Although n-3 and n-6 series fatty acids alter the availability of cholesterol as a substrate for progesterone synthesis, the most important effect of these acids appears to be their capacity to regulate the synthesis of PGF_2α_ and the subsequent effect of prostaglandins on progesterone synthesis [3]. However, due to the complex mechanisms involved in the synthesis of prostaglandins from fatty acids, the results of studies linking dietary PUFA with prostaglandin synthesis and their effects on reproduction in cattle are variable and inconsistent. Most in vitro studies show that n-3 supplementation down-regulates the production of prostaglandins, while the opposite is true of n-6. However, very few in vivo studies have been able to associate changes in prostaglandin synthesis with reproductive results.

The higher concentration of NEFA in the oil-supplemented diets (SOY and LIN) observed in our study are similar to those reported by Grummer and Carroll [56]. In this data review, the authors report that plasma NEFA concentration is usually higher in cows supplemented with fats compared to diets without supplementation, due to an increased release of fatty acids from circulating lipoprotein triglycerides. Similarly, Johnson et al. [57] observed a trend toward a higher concentration of NEFA in cows receiving rations supplemented with 4.0% or 5.6% oilseed fat when compared to the control diet (no added fat). Other studies, in contrast, observe a decrease [58,59] or no difference [11,52] in plasma NEFA concentrations in fat-supplemented diets. However, these studies compared diets with PUFA-rich oils against iso-lipid diets containing saturated fat, while we compared a diet with no added fat against PUFA-rich diets.

The IGF-1 levels observed in our study differ from those observed by Robinson et al. [60] in Holstein cows receiving a control diet, a diet with soybean oil, and a diet with linseed oil. In the foregoing study, mean IGF-1 levels were significantly higher in cows fed rations with soybean oil compared to the control diet or linseed oil. Our data, in contrast, show higher levels in the control diet, followed by the SOY diet, with the lowest levels observed in the LIN diet. Caldari-Torres et al. [59] also observed higher plasma IGF-1concentrations in seven-week-postpartum Holstein cows fed with oil rich in sunflower oil compared with cows that consumed a control ration enriched with saturated fatty acids. In contrast to our results, Taylor et al. [61] observed that cows with higher plasma IGF-1 concentrations and a positive energy balance during the first 12 weeks postpartum were more likely to become pregnant than those with low IGF-1 levels. However, Bilby et al. [62] found evidence of an IGF-I concentration threshold that is associated with increased fertility, and showed that increasing IGF-I levels above this threshold could have a negative impact on the gestation rate.

Reis et al. [63], which is in line with our results, observed no differences in insulin concentrations between a diet with no added fat and a diet supplemented with Ca salts of PUFA. However, Bilby et al. [62], in a study supplementing rations with Ca salts of PUFA, and Choi and Palmquist, [64] giving increasing amounts of long chain fatty acids, observed a reduction in plasma insulin levels. Similarly, Staples et al. [2], in a literature review, reported that plasma insulin decreased in seven out of nine studies that supplemented rations with fat. Other studies, in contrast, show increased insulin levels when rations are supplemented with Ca salts of PUFA [65], while Van Knegsel et al. [66], in a literature review, report that supplementation with extralipogenic nutrients, such as fats, generally increases NEFA and BHBA levels and decreases insulin levels. In addition, diets with a higher content of glycogenic nutrients, such as non-fibrous carbohydrates, increase insulin levels and decrease the plasma NEFA and BHBA concentrations. According to these authors, the effects of supplementary fat depend largely on the nature of the other nutrients in the diet and on the ruminal fermentation pattern. In our study, the CON diet contained no added fat. Therefore, in order for it to be isoenergetic with the oil-rich LIN and SOY diets, it contained a greater amount of non-fibrous carbohydrates (starch), and, therefore, the pattern of ruminal fermentation differs with respect to the LIN and SOY diets. Many of the differences observed in blood parameters are probably due to differences in the type of diet consumed by the animals. Most studies on the effects of PUFA supplementation use control diets supplemented with saturated fat, but very few studies compare PUFA supplementation with a control diet that provides the same energy and protein levels without added fat. This could explain the differences between our results and those reported by other authors.

## 5. Conclusions

In conclusion, the present experiment showed that the addition of 2.3% of oils rich in PUFA to TMR does not affect milk yield, reduces its fat and protein content, increases the content of C18:2cis-9, trans 11 CLA, MUFA, PUFA, and n-3 PUFA, and reduces the levels of SMCFA, SFA, and the atherogenicity index, which greatly improves their nutritional profile.

Feeding dairy cows with n-3 PUFA helps increase the number of pregnant cows to first TAI and reduce the number of TAI, but these benefits were not enough to statistically increase the number of pregnant cows.

## Figures and Tables

**Table 1:** Ingredients and estimated nutritive value of the experimental diets.

Item	Diet ^1^		
	CON	SOY	LIN
Ingredients (g/kg dry matter basis)			
Corn silage	181.0	186.0	186.0
Alfalfa hay	204.3	209.0	209.0
Barley straw	19.6	20.3	20.3
Soy hulls	57.2	58.8	58.8
Barley grain, ground	28.5	162.7	162.7
Wheat grain, ground	179.9	78.6	78.6
DDGS ^2^	100	-	-
Palm kernel cake	54.9	40.2	40.2
Soybean meal	52.4	96.4	96.4
Barley sprouts	38.5	39.6	39.6
Wheat bran	52.6	54.0	54.0
Molasses, sugarcane	13.3	13.6	13.6
Soybean oil	-	23.0	-
Linseed oil	-	-	23.0
Limestone	4.0	4.0	4.0
Dicalcium phosphate	0.8	0.8	0.8
Sodium bicarbonate	9.6	9.6	9.6
Sodium chloride	1.7	1.7	1.7
Mineral and vitamin mix ^3^	1.7	1.7	1.7
Estimated nutritive value			
UFL ^4^	0.95	0.95	0.95
PDI ^5^	104.0	104.0	104.4

^1^ CON = control group with no fat supplementation; SOY = fat supplementation based on soybean oil; LIN = fat supplementation based on linseed oil. ^2^ Dried distillers grains with soluble. ^3^ Mineral-vitamin mix (De Heus Nutrición Animal, La Coruña, Spain) provided (per kg of premix): Vitamin A 3,000,000 UI, Vitamin D3 750,000 UI, Vit E 12,500 UI, zinc oxide 20,500 mg, zinc chelate 7500 mg, cupric sulphate 5500 mg, cupric chelate 750 mg, sodium selenite 200 mg, Calcium 1904 mg, Sodium 222 mg. ^4^ UFL: feed unit for milk production (UFL/kg DM estimated from INRA 2007) ^5^ PDI: protein truly digestible in the small Intestine (g/kg DM estimated from INRA 2007). LIN = fat supplementation based on linseed oil.

**Table 2:** Chemical and fatty acid composition of the experimental diets.

Item	Diet ^1^		
	CON	SOY	LIN
Chemical composition (g/kg DM)			
Dry matter	670.0	637.2	626.8
Crude protein	171.1	163.9	163.5
Ether extract	36.2	49.8	48.8
Neutral detergent fibre	306.8	314.5	329.5
Acid detergent fibre	219.4	234.0	252.1
Starch	272.0	228.1	217.9
Fatty acids (% of total fatty acids)			
C12:0	5.23	2.37	4.18
C14:0	1.82	1.06	1.6
C16:0	25.05	20.32	18.32
C16:1	1.3	0.82	0.53
C17:0	0.32	0.29	0.21
C17:1	0.32	0.11	0.21
C18:0	4.72	3.46	4.36
C18:1	17.54	18.68	17.55
C18:2	32.75	42.56	28.3
C18:3	9.6	8.26	24.10
C20:0	1.17	0.41	0.39
C20:1	0.18	1.66	0.25
Saturated fatty acids	38.31	27.91	29.06
Monounsaturated fatty acids	19.34	21.27	18.54
Polyunsaturated fatty acids	42.35	50.82	52.41

^1^ CON = control group with no fat supplementation, SOY = fat supplementation based on soybean oil, LIN = fat supplementation based on linseed oil. (Aceites de semillas S.A., Barcelona Spain).

**Table 3:** Dry matter intake, milk yield, and milk composition.

Item	Diet ^1^			SEM ^2^	*p*-Value
	CON	SOY	LIN		
Milk YUield (kg/d)	31.1	32.0	31.6	1.02	0.806
4% fat-corrected milk yield (kg/d)	28.1	26.2	25.8	1.51	0.556
Fat (%)	3.41 ^a^	2.65 ^b^	2.99 ^b^		0.011
Fat Yield (kg/d)	1.06	0.88	0.89	0.068	0.098
Protein (%)	3.33 ^a^	3.09 ^b^	3.16 ^b^	0.062	0.022
Protein Yield (kg/d)	0.98	1.00	0.98	0.061	0.921

^1^ CON = control group with no fat supplementation, SOY = fat supplementation based on soybean oil, LIN = fat supplementation based on linseed oil; ^2^ SEM = standard error of the mean, ^a,b^ values within a row with different superscripts differ significantly at *p* < 0.05.

**Table 4:** Fatty acids composition of milk (g/100 g total fatty acids).

Item	Diet ^1^			SEM ^2^	*p*-Value
	CON	SOY	LIN		
Saturated fatty acids (SFA)	67.84 ^a^	60.75 ^b^	62.02 ^b^	0.951	<0.0001
C4:0	1.22 ^a^	0.942 ^b^	1.05 ^b^	0.049	0.002
C8:0	0.085	0.177	0.074	0.0441	0.222
C12:0	3.99 ^a^	2.76^c^	3.62 ^b^	0.331	0.039
C14:0	13.65	13.33	12.89	0.528	0.611
C16:0	41.64 ^a^	34.86 ^b^	34.53 ^b^	0.383	<0.0001
C18:0	7.26 ^b^	8.98 ^a^	9.80 ^a^	0.467	0.002
Monounsaturated fatty acids (MUFA)	26.14 ^b^	32.01 ^a^	30.17 ^a^	0.859	<0.001
C14:1	1.69 ^a^	1.59 ^b^	1.39 ^c^	0.068	0.019
C16:1	2.83 ^a^	2.343 ^b^	2.12 ^b^	0.172	0.023
C18:1 trans-11	1.55 ^c^	1.80 ^b^	3.45 ^a^	0.230	<0.0001
C18:1 cis-9	20.06 ^c^	26.24 ^a^	23.20 ^b^	0.933	0.004
Polyunsaturated fatty acids (PUFA)	5.72 ^b^	7.01 ^a^	7.40 ^a^	0.201	<0.0001
C18:2 cis-9.cis-12n-6	3.99 ^c^	5.01 ^a^	4.51 ^b^	0.118	<0.0001
C18:2 cis-9. trans-11 CLA	0.58 ^c^	0.82 ^b^	1.30 ^a^	0.083	<0.0001
C18:2 trans-10. cis-12 CLA	0.064 ^c^	0.128 ^a^	0.088 ^b^	0.0102	<0.0001
C18:3 cis-9. cis-12. cis-15n-3	0.640 ^c^	0.653 ^b^	1.11 ^a^	0.048	<0.0001
C20:4 n-6	0.239 ^a^	0.164 ^b^	0.170 ^b^	0.0271	0.090
C20:5 n-3	0.138	0.160	0.167	0.0160	0.208
PUFA/SFA	0.085 ^b^	0.116 ^a^	0.121 ^a^	0.0043	<0.0001
n-3 PUFA	0.845 ^c^	0.891 ^b^	1.34 ^a^	0.051	<0.0001
n-6 PUFA	4.23 ^c^	5.17 ^a^	4.67 ^b^	0.118	<0.0001
n6/n3	5.34 ^ab^	6.04 ^a^	3.63 ^b^	0.266	<0.0001
Short and medium chain fatty acids (SMCFA) ^3^	65.10 ^a^	55.76 ^b^	55.77 ^b^	1.019	<0.0001
Long chain fatty acids (LCFA) ^4^	34.60 ^b^	44.07 ^a^	43.83 ^a^	1.021	<0.0001
∆^9^ Desaturase index					
C14:1/(C14:0 + C14:1)	0.119 ^ab^	0.122 ^a^	0.107 ^b^	0.004	0.090
C16:1/(C16:0 + C16:1)	0.063	0.063	0.058	0.0040	0.567
C18:1/(C18:0 + C18:1)	0.744 ^ab^	0.741 ^a^	0.706 ^b^	0.0212	0.053
C18:2 cis-9. trans-11/(C18:1 trans-11 + C18:2 cis-9. trans-11)	0.289 ^ab^	0.393 ^a^	0.268 ^b^	0.0331	0.031
Atherogenicity index ^5^	3.29 ^a^	2.38 ^b^	2.42^b^	0.163	<0.0001

^1^ CON = control group with no fat supplementation, SOY = fat supplementation based on soybean oil, LIN = fat supplementation based on linseed oil; ^2^ SEM = standard error of the mean; ^3^ C4 to C16; ^4^ ≥C18; ^5^ (C12:0 + 4 × C14:0 + C16:0)/(MUFA + PUFA) (Ulbricht and Southgate. 1991); ^a.b.c^ values within a row with different superscripts differ significantly at *p* < 0.05.

**Table 5:** Fertility response of cows.

Item	Diet ^1^			*p*-Value
	CON (*n* = 65)	SOY (*n* = 64)	LIN (*n* = 65)	
Number of pregnant to first TAI	15 ^b^	23 ^ab^	27 ^a^	0.07
Number of pregnant	46	48	51	0.60
Number of TAI ^2^ Median (interquartile range)	3 (2) ^a^	2(3) ^ab^	2(3) ^b^	0.08
Open days ^3^ Median (interquartile range)	164 (100)	155 (102)	123 (89)	0.29

^1^ CON = control group with no fat supplementation, SOY = fat supplementation based on soybean oil, LIN = fat supplementation based on linseed oil; ^2^ TAI = fixed-time artificial insemination; ^3^ Days from calving to confirmed pregnancy; ^a,b^ values within a row with different superscripts differ significantly at *p* < 0.05.

**Table 6:** Progesterone and metabolites concentration in plasma.

Item	Diet ^1^			Mean	SEM ^2^	*p*-Value		
	CON	SOY	LIN			D	T	DXT
**Progesterone (ng/mL)**						0.09	<0.0001	0.12
Time 1	4.24	4.77	5.39	4.80				
Time 2	0.67	0.77	0.73	0.73				
Time 3	4.39	4.64	5.97	5.00				
Mean	3.10 ^b^	3.40 ^ab^	4.03 ^a^		0.311			
**BHBA (mmol/L)**						0.31	<0.0001	0.30
Time 1	0.51	0.50	0.53	0.51				
Time 2	0.38	0.40	0.46	0.42				
Time 3	0.52	0.50	0.52	0.51				
Mean	0.47	0.47	0.50		0.018			
**NEFA (mmol/L)**						0.04	<0.0001	0.45
Time 1	0.49	0.55	0.49	0.51				
Time 2	0.24	0.30	0.29	0.28				
Time 3	0.20	0.24	0.27	0.24				
Mean	0.31 ^b^	0.36 ^a^	0.35^a^		0.015			
**Insulin ((µg/L)**						0.63	<0.0001	0.28
Time 1	0.74	0.70	0.67	0.70				
Time 2	0.49	0.53	0.50	0.50				
Time 3	0.89	0.73.	0.76	0.79				
Mean	0.71	0.65	0.64		0.049			
**IGF-1 (ng/mL)**						0.002	<0.0001	0.63
Time 1	157.47	137.45	118.8	137.91				
Time 2	159.82	155.10	135.73	150.22				
Time 3	167.83	154.05	137.02	153.02				
Mean	161.89 ^a^	149.02 ^b^	130.60 ^c^		6.25			

^1^ CON = control group with no fat supplementation, SOY = fat supplementation based on soybean oil. LIN = fat supplementation based on linseed oil; ^2^ SEM = standard error of the mean; ^a,b,c^ values within a row with different superscripts differ significantly at *p* < 0.05.
